# Omics approach to chest electrical impedance tomography reveals physiological cluster of ARDS characterised by increased respiratory drive and effort

**DOI:** 10.1186/s13613-025-01514-3

**Published:** 2025-07-08

**Authors:** Tommaso Mauri, Marco Leali, Elena Spinelli, Gaetano Scaramuzzo, Massimo Antonelli, Domenico L. Grieco, Savino Spadaro, Giacomo Grasselli

**Affiliations:** 1 Department of Pathophysiology and Transplantation, University of Milan, Via F. Sforza 35, Milan, 20122 Italy; 2 Department of Emergency, Foundation IRCCS Ca’ Granda Maggiore Policlinico Hospital, Milan, Italy; 3 Department of Translational Medicine, University of Ferrara, Ferrara, Italy; 4 Intensive Care Unit, Department of Morphology, Surgery and Experimental Medicine, Sant’Anna University Hospital, Ferrara, Italy; 5 Department of Emergency, Intensive Care Medicine and Anesthesia, Fondazione Policlinico Universitario A. Gemelli IRCCS, Rome, Italy; 6 Department of Anesthesiology and Intensive Care Medicine, Catholic University of the Sacred Heart, Rome, Italy

**Keywords:** Omics, Hierarchical clustering, Clustering, Agglomerative nesting, ARDS, Electrical impedance tomography, EIT, Esophageal pressure, Respiratory drive, V’/Q

## Abstract

**Background:**

Non-invasive assessment of respiratory drive and effort in spontaneously breathing ARDS patients is challenging, yet clinically relevant. We explored whether hierarchical clustering applied to electrical impedance tomography (EIT– a radiation-free non-invasive lung imaging technique) identifies ARDS sub-phenotypes with increased drive and effort.

**Results:**

Thirty intubated patients with ARDS on assisted mechanical ventilation were monitored by EIT and esophageal pressure during a decremental positive end-expiratory pressure (PEEP) trial. A comprehensive EIT assessment was made (computed variables n = 180) during tidal breathing at different PEEP levels. Agglomerative nesting was applied to scaled data distances. Three clusters of ARDS were identified: *inhomogeneous ventilation*, *unmatched V’/Q*, and *mismatched V’/Q*. The *unmatched V’/Q* cluster had the highest respiratory drive (*p* = 0.045) and effort (*p* = 0.021) at lower PEEP, and experienced longer length of ICU stay (*p* = 0.019).

**Conclusions:**

Higher PEEP levels reduced drive of the *unmatched V’/Q* cluster, mitigating the physiological differences. Clustering approaches to EIT data identify physiologically and clinically relevant sub-phenotypes of ARDS.

**Supplementary Information:**

The online version contains supplementary material available at 10.1186/s13613-025-01514-3.

## Background

The acute respiratory distress syndrome (ARDS) is characterized by rapid development of hypoxemia and bilateral pulmonary infiltrates on chest x-ray, with preserved cardiac function. Diagnosis of ARDS often require prolonged mechanical ventilation and stay in the intensive care unit, have very high mortality, and specific treatments are still missing [1, 2].

Physiological characterization of ARDS could help to identify specific sub-phenotypes benefiting from personalised treatments. Spontaneously breathing ARDS presenting with increased respiratory drive and effort are at risk for patient self-inflicted lung injury (P-SILI), which could worsen ARDS and increase mortality, and might benefit from personalised titration of positive end-expiratory pressure (PEEP) [3, 4].

Electrical impedance tomography (EIT) is a bedside, radiation-free, non-invasive, continuous monitor of regional pulmonary ventilation and perfusion and of their matching (V’, Q and V’/Q) [5, 6]. EIT data from ARDS allows calculations of multiple physiological indexes, which quantify regional derangements of V’, Q and V’/Q mismatch and were associated with with increased risk of worsening lung injury. To date, most studies performed with EIT on ARDS patients focused on single or very few physiological indexes. Some sought for identification of sub-phenotypes [7] but, to our knowledge, never in spontaneously breathing patients and never looking for a correlation between EIT data and increased respiratory drive [8, 9].

In the present hypothesis-generating study, we tested a novel approach to EIT, simultaneously analysing 180 physiological indexes to identify physiological clusters of ARDS; secondly, we verified whether these clusters presented different respiratory drive, effort and clinical outcomes; third, we explored the potentiality of EIT to assign a personalised protective PEEP level.

## Method

### Clustering of patients

We analysed data from 30 patients with ARDS, intubated and spontaneously breathing, enrolled from 3 Italian academic ICUs (Intensive Care Units) [4]. The protocol was approved by the Ethical Committees of each centre and informed consent was obtained. Patients were aged 64 ± 14, their mean P/F ratio was 205 ± 55 mmHg and they were mostly affected by infectious (22/30, 73%) and primarily pulmonary (22/30, 73%) ARDS. A detailed patient description can be found in the primary paper [4] and in Table 1.

**Table 1:** Baseline patient characteristics

	***PEEPlow***				
	**ALL** **(** ***n*** **= 30)**	**UNMATCHED** **(** ***n*** **= 15)**	**MISMATCHED** **(** ***n*** **= 9)**	**INHOMOG. VENTILATION** **(** ***n*** **= 6)**	***p*** **-value**
age (years)	64 ± 14	59 ± 15	68 ± 12	71 ± 12	0.178
gender (male / female)	23 / 7 (77% / 13%)	12 / 3 (80% / 20%)	7 / 2 (78% / 22%)	4 / 2 (67% / 33%)	0.805
BMI (kg/m^2)	28 ± 5	28 ± 6	27 ± 3	27 ± 5	0.944
P/F (mmHg)	205 ± 55	198 ± 58	202 ± 43	228 ± 66	0.515
PaCO2 (mmHg)	45 ± 6	47 ± 5	43 ± 7	43 ± 6	0.239
pH	7.44 ± 0.04	7.43 ± 0.04	7.44 ± 0.06	7.43 ± 0.03	0.914
Ventilatory Ratio	1.69 ± 0.46	1.70 ± 0.49	1.58 ± 0.29	1.81 ± 0.62	0.642
Vt/PBW (ml/kg)	7.88 ± 1.8	7.75 ± 1.46	8.57 ± 2.19	7.19 ± 1.94	0.333
Pulmonary ARDS	22 (73%)	12 (80%)	7 (77.78%)	3 (50%)	0.350
Infectious ARDS	22 (73%)	11 (73.33%)	8 (88.89%)	3 (50%)	0.249
	***PEEPintermediate***				
	**ALL** **(** ***n*** **= 30)**	**UNMATCHED** **(** ***n*** **= 7)**	**MISMATCHED** **(** ***n*** **= 11)**	**INHOMOG. VENTILATION** **(** ***n*** **= 12)**	***p*** **-value**
age (years)	64 ± 14	63 ± 18	66 ± 8	63 ± 17	0.887
gender (male / female)	23 / 7 (77% / 13%)	5 / 2 (71% / 29%)	10 / 1 (91% / 9%)	8 / 4 (67% / 33%)	0.363
BMI (kg/m^2)	26.64 (24.61–30.86)	26 (25–29)	27 (26–31)	27 (23–31)	0.638
P/F (mmHg)	205 ± 55	227 ± 50	178 ± 47	217 ± 58	0.110
PaCO2 (mmHg)	45 ± 6	44 ± 5	46 ± 8	45 ± 5	0.751
pH	7.44 ± 0.04	7.43 ± 0.04	7.46 ± 0.04	7.41 ± 0.04	0.022
Ventilatory Ratio	1.61 (1.38–1.87)	1.48 (1.32–1.65)	2.00 (1.73–2.26)	1.46 (1.27–1.65)	0.017
Vt/PBW (ml/kg)	7.57 (6.66–8.54)	7.72 (6.8–8.07)	7.23 (6.74–8.3)	8.04 (6.52–9.9)	0.786
Pulmonary ARDS	22 (73%)	5 (71.43%)	10 (90.91%)	7 (58.33%)	0.209
Infectious ARDS	22 (73%)	5 (71.43%)	7 (63.64%)	10 (83.33%)	0.561
	***PEEPhigh***				
	**ALL** **(** ***n*** **= 28)**	**UNMATCHED** **(** ***n*** **= 6)**	**MISMATCHED** **(** ***n*** **= 15)**	**INHOMOG. VENTILATION** **(** ***n*** **= 7)**	***p*** **-value**
age (years)	64 ± 14	62 ± 19	64 ± 14	61 ± 11	0.910
gender (male / female)	23 / 7 (77% / 13%)	5 / 1 (83% / 17%)	13 / 2 (87% / 13%)	5 / 2 (71% / 29%)	0.683
BMI (kg/m^2)	26.64 (25.04–30.86)	26 (25–27)	26 (25–30)	29 (27–33)	0.411
P/F (mmHg)	205 ± 55	233 ± 51	199 ± 57	204 ± 58	0.473
PaCO2 (mmHg)	45 ± 6	44 ± 6	46 ± 7	43 ± 4	0.534
pH	7.44 ± 0.05	7.45 ± 0.04	7.45 ± 0.04	7.39 ± 0.05	0.015
Ventilatory Ratio	1.61 (1.38–1.93)	1.53 (1.40–1.69)	1.76 (1.47–2.17)	1.43 (1.30–1.65)	0.434
Vt/PBW (ml/kg)	7.57 (6.66–8.56)	7.78 (7.32–8.19)	6.87 (6.55–8.3)	8.51 (7.57–9.94)	0.251
Pulmonary ARDS	21 (75%)	3 (50%)	14 (93.33%)	4 (57.14%)	0.053
Infectious ARDS	21 (75%)	5 (83.33%)	11 (73.33%)	5 (71.43%)	0.864

Baseline patient characteristics for each cluster at each PEEP level are shown. ARDS– acute respiratory distress syndrome; BMI– body mass index; INHOMOG.– inhomogeneous; PaCO2– arterial tension of carbon dioxide; PBW– predicted body weight; PEEP– positive end-expiratory pressure; P/F– ratio between the arterial oxygen tension and the fraction of inspired oxygen; pH– negative logarithmic concentration of hydrogen ions; Vt– tidal volume

**Fig. 1:**
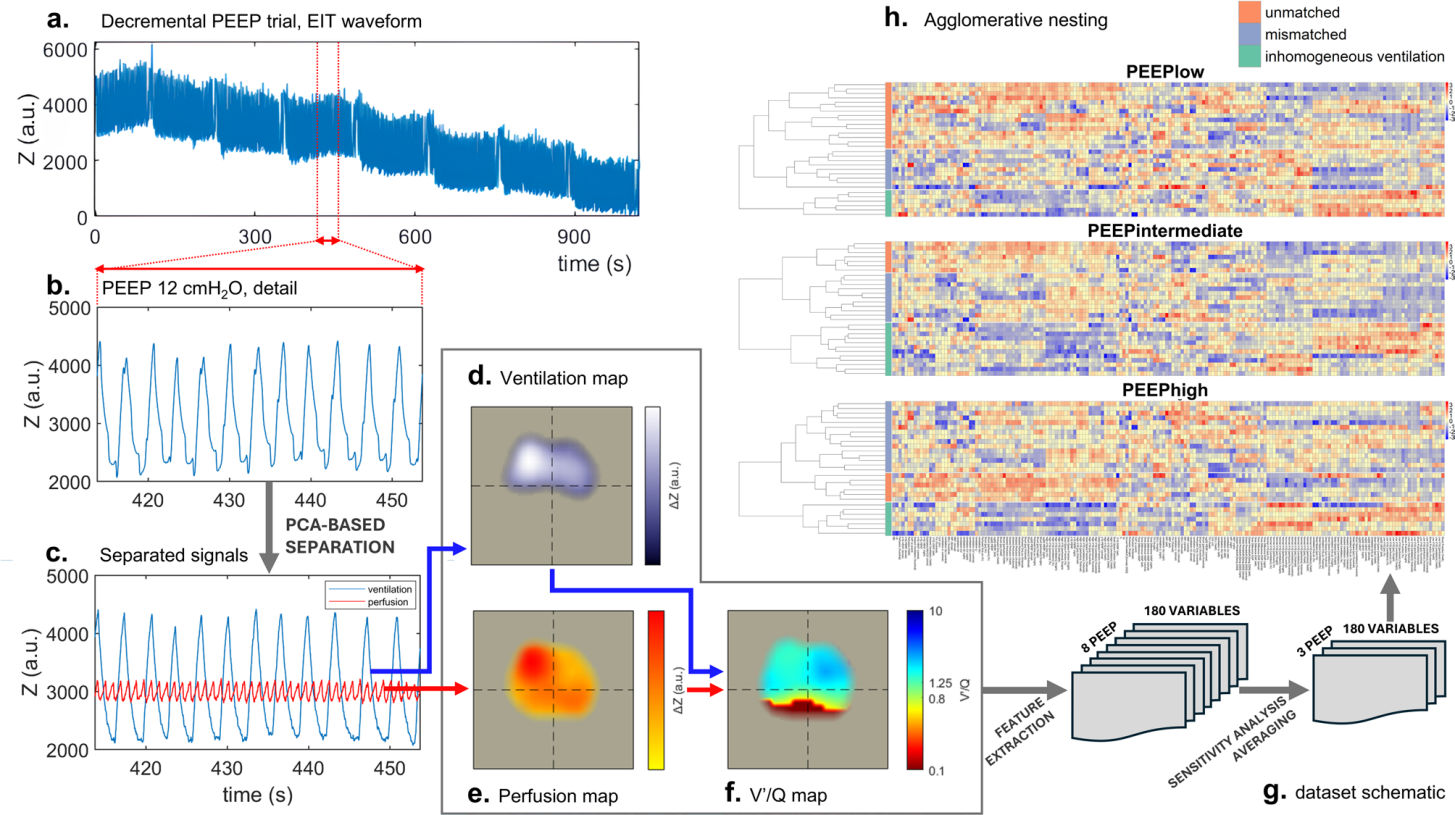
EITomics approach and identification of ARDS clusters. Panels **a**-**f**. Show data from one representative study patient. Panels **a**-**b**. EIT waveform from positive end-expiratory pressure (PEEP) trial ranging from 18 to 4 cmH_2_O, with 2 cmH_2_O decrease every 2 minutes. Panels **c**-**e**. A previously published algorithm [12] based on principal component analysis (PCA) was used to separate the ventilation-related from the cardiac-related EIT signal components at each step, which were used to create EIT ventilation (**d**) and perfusion (**e**) maps, respectively. Panel **f**. By superimposition [30]V’/Q maps were generated. Panel **g**. PEEP steps were averaged to 3 main levels (PEEPlow, PEEPintermediate and PEEPhigh), after conducting a sensitivity analysis based on the agglomerative coefficient. Panel **h**. Heatmaps and dendrograms representing the agglomerative nesting process. Columns represent variables, rows represent patients. Blank spaces have been added between patient clusters. Standardized data (z-scores) are color-coded into the heatmaps. Three clusters were identified at each PEEP level, named *unmatched V’/Q*, *mismatched V’/Q* and *inhomogeneous ventilation*

While on pressure support ventilation, patients underwent a decremental PEEP trial (ranging from 18 to 4 cmH2O in steps of 2 cmH2O, lasting 2 minutes each), with EIT and transpulmonary pressure monitoring [4] (Fig. 1a). Light sedation, support (8 ± 2 cmH2O) and FiO2 (45 ± 8%) were kept constant [4]. At each step and for each patient a comprehensive assessment of EIT variables was made, comprising 64 variables from regional ventilation and perfusion (pulsatility) plus 116 variables from regional ventilation/perfusion (pulsatility) matching (V’/Q) [10], for a total of 180 variables. (see the complete list in Table S1). Variables were computed from EIT regional ventilation and perfusion maps during tidal breathing (Fig. 1b-f). While indicator/saline–based perfusion during uninterrupted breathing is currently being experimented [11] it is not currently feasible during spontaneous breathing and was not part of our protocol. Pulsatility was therefore extracted as previously described [12]. For the sake of simplicity, we will refer to it as perfusion in the remaining part of the paper, as, to our knowledge and up to date, it is the only available proxy for perfusion in this type of EIT recordings.

Hierarchical clustering was chosen as an unsupervised clustering methodology which relies on few if any assumption [13] and which is widely applied in the biomedical field [14]. No prior knowledge of the number of clusters is required and dendrograms and heatmaps can be used to easily visualize the clustering process and features [13, 14]. These characteristics make it well-suited for our analysis, whose main focus is on feasibility and data exploration.

The first step of any hierarchical clustering algorithm is to calculate a dissimilarity (or similarity) metric for each couple of items to be clustered: this can be easily understood as the generalization of distance calculated over all the dimensions (i.e. variables) of a dataset. In our study, the dissimilarity metric was the Euclidean distance in a 180-dimensional space.

The second step is to cluster items in by a hierarchical method. In our implementation, an algorithm called AGNES (agglomerative nesting) was used: it starts by merging the two closest individuals/items and then progresses merging the closest couple of individuals or clusters until all of them have been merged into a single cluster. This process can be easily visualized as a dendrogram (Fig. 1h). To perform this process, one must define the concept of “distance between clusters” soon after merging the first couple of items: this is called the “linkage method”. Any choice about how to define similarity between clusters can be debated, and we performed a sensitivity analysis to choose among the several available options (see below).

Finally, one should choose how to “cut” the dendrogram, i.e. where the algorithm must stop obtaining a definite number of clusters before merging all items in one single cluster. The choice of our stopping rules is further detailed in the Supplementary Methods section.

Excellent books have been written on hierarchical clustering and similar techniques: Kaufman & Rousseeuw’s work has served us as a reference [13].

Hierarchical clustering was performed at the patient level, treating different levels of PEEP separately, i.e. within each heatmap/dendrogram each patient was subject to the same level of PEEP. Before calculating the dissimilarity matrix, variables were pre-processed as suggested [13]: ratio-scaled variables were log-transformed, then z-scores were calculated. A dissimilarity matrix was built by using Euclidean distances among patients. The AGNES algorithm was then applied to patient data [13].

To improve the signal-to-noise ratio of our EIT metrics and to facilitate readability and interpretation of our results, we averaged consecutive PEEP levels. We compared different possible PEEP groupings in a sensitivity analysis, which also compared the effect of different linkage methods to define between-cluster distances (Fig. 1g, Table S2). The sensitivity analysis was based on the agglomerative coefficient, a metric measuring the strength of grouping (for further details, see Supplementary Methods) which accounts also for between patient variability.S1.

The whole clustering process was unsupervised, i.e. blind with respect to physiological variables of respiratory drive and effort and to baseline patient characteristics.

### Feature extraction

Features differing significantly among clusters were selected by applying a one-way analysis of variance (ANOVA) or Kruskal-Wallis test to each feature and correcting the resulting *p*-value with the Bonferroni rule. For each feature, intra-cluster consistency was assessed by calculating the intra-class correlation coefficient (ICC) assuming a one-way random model. This is also known as ICC(1 A,1) under a popular naming convention [15, 16]. The unequal number of subjects within clusters was handled as in [15]. According to existing guidelines, ICC values above 0.6 indicate good agreement and values above 0.75 indicate excellent agreement [17]. A 99% confidence level was used for selecting significant features.

### External validation

In order to verify whether the obtained clustering partition reflects patient physiology and characteristics, hard outcomes and patient physiology, as obtained by specialized measurements less commonly available in the ICU and relatively more invasive than EIT, were compared between clusters. These measurements have been performed as part of the original study protocol [4]. The difference between end-inspiratory and end-expiratory esophageal pressure (∆Pes) and transpulmonary pressure (∆Plung) have been assessed during spontaneous breathing over 10–15 breaths by an experienced operator, who also assessed the drop in airway pressure during the first 100 ms of an end-expiratory occlusion, as the gold standard method to measure p0.1, an index of respiratory drive [18]. The PEEP level suggested by integrating esophageal pressure and EIT measurements (PEEPeit) has been obtained according to a previously published algorithm [19]. Finally, the length of ICU stay had been previously recorded as an hard outcome.

None of these measurements has been employed during the clustering process. Although PEEPeit makes use of EIT data, it is also influenced by respiratory effort and none of the metrics used to determine it (i.e. lung overdistension and collapse) has been included in the clustering analysis.

### Statistics

Comparison of continuous variables across clusters was performed with one-way analysis of variance (ANOVA) or Kruskal-Wallis test, as appropriate. The normality of residuals was assessed by the Shapiro-Wilk test, homoscedasticity by the Levene test. Post-hoc tests were Tukey’s honestly significant difference (HSD) in the parametric case, Dunn’s test with Bonferroni correction in the non-parametric case. Differences in qualitative variables across clusters were assessed with the chi-square test. A 95% confidence level was adopted.

### Software

MatLab R2022a was used for EIT data processing and feature extraction. R software [20] version 4.2.1 was used for clustering and statistical computations. In particular, package *NbClust* was used for internal validation rules used to determine the number of clusters [21].

## Results

Baseline patient characteristics are shown in Table 1. Sensitivity analysis (Fig. 1g, Table S2) showed that the strongest clustering structure was found by using Ward’s linkage criterion and by averaging values of each physiological variable obtained during the following PEEP steps: 4-6-8 cmH2O (PEEPlow, AC = 0.65), 10-12-14 cmH2O (PEEPintermediate, AC = 0.72) and 16–18 cmH2O (PEEPhigh, AC = 0.69). These adjacent PEEP levels were averaged, treating them similarly to replicates. Patient clustering was then performed separately within each of the resulting three PEEP levels.

**Fig. 2:**
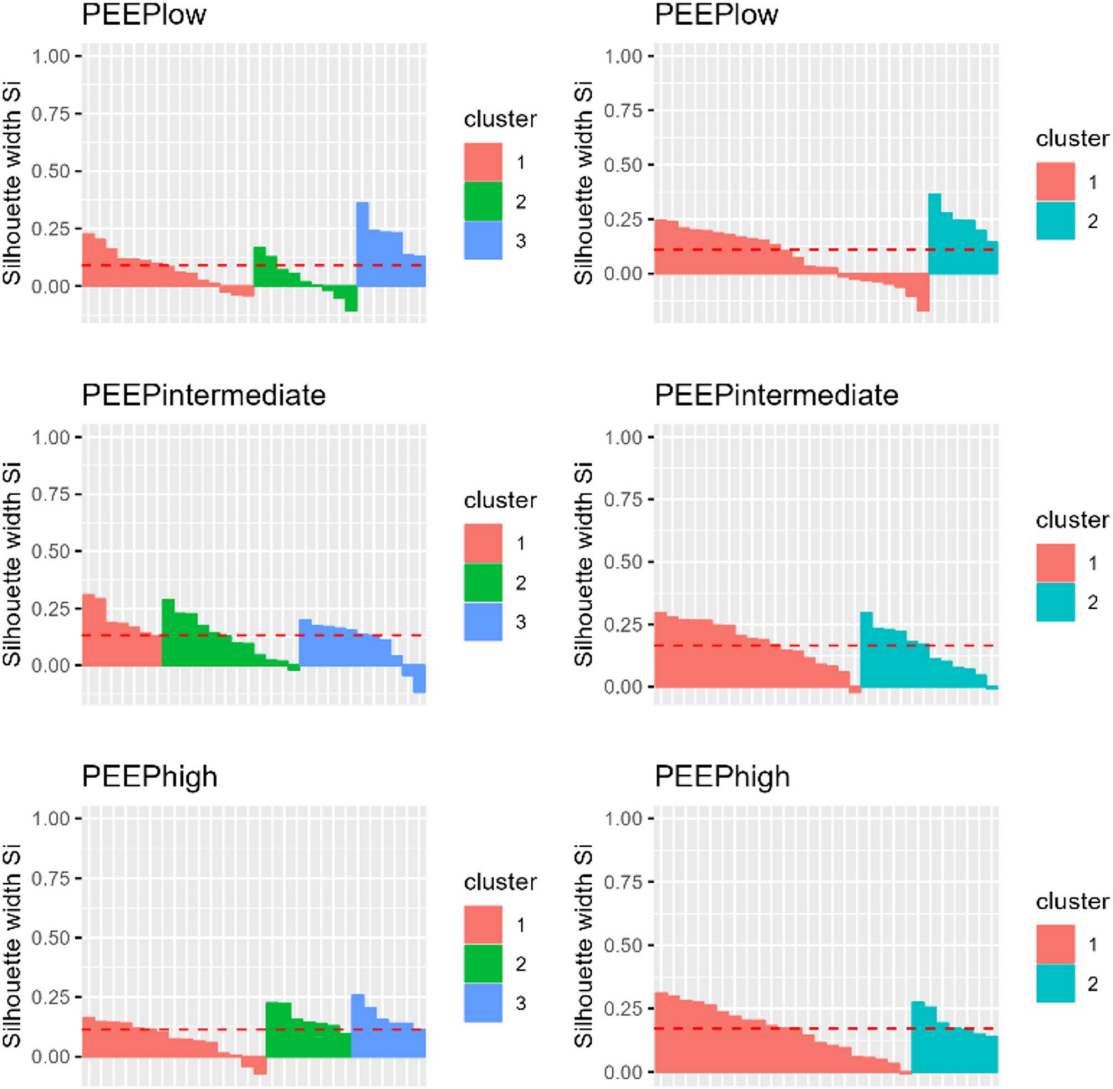
Silhouette plots for selecting the number of clusters. In a silhouette plot, each subject is represented by a bar: the higher the bar, the lower is the average distance of that subject from the other subjects within the same cluster, as compared to its distance from the next closest cluster. Negative bars indicate misclassified subjects based on this criterion [31]. The average bar height is marked by a red dotted line and is thought to represent an index of the strength of the clustering.Plots under the hypothesis of two or three clusters are compared and used as an aid for selecting the number of clusters, in addition to the above mentioned stopping rules (see section Methods). The choice of three clusters resulted in fewer misclassified patients at PEEPlow

**Fig. 3:**
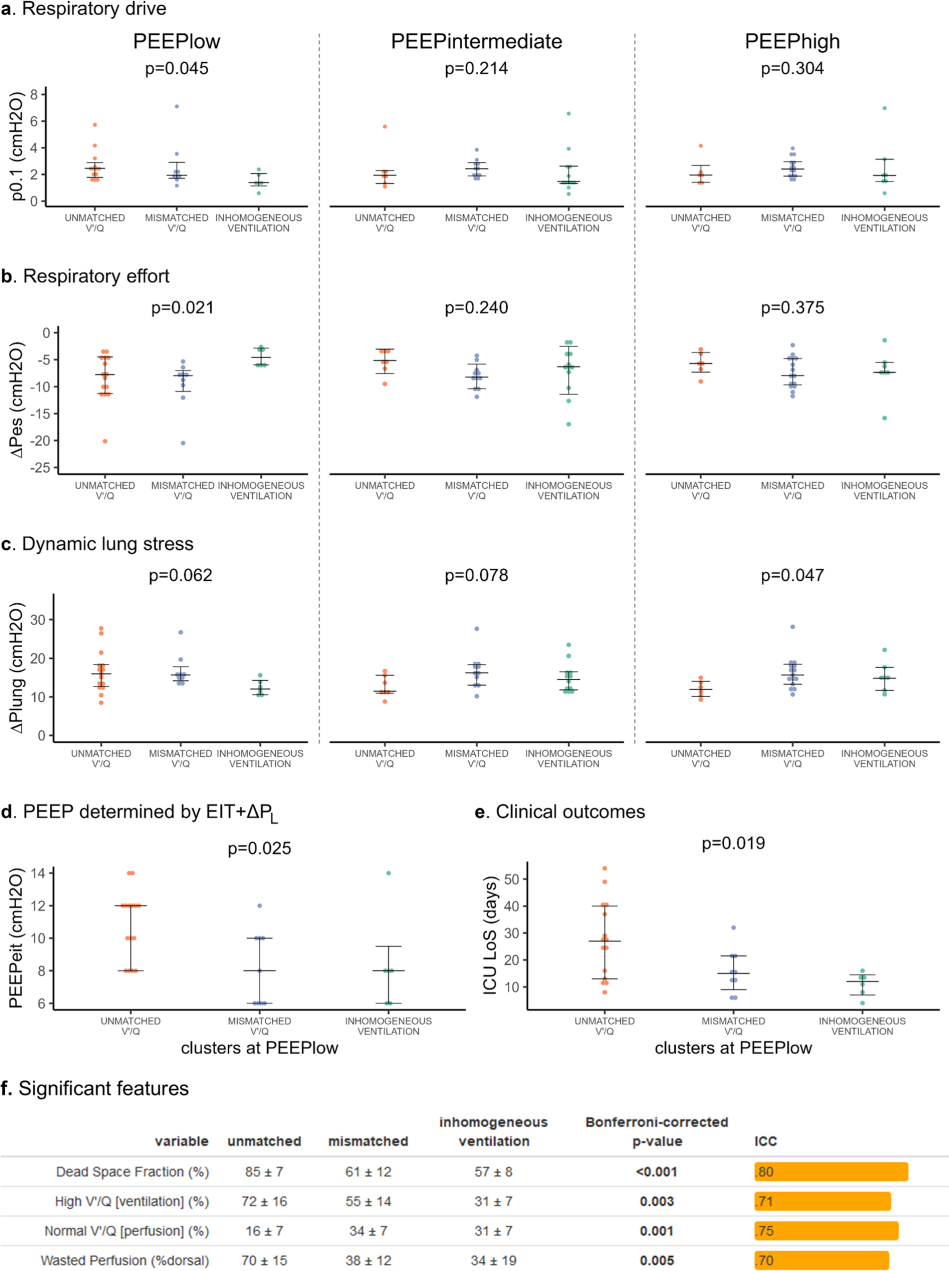
Respiratory drive and effort, clinical outcome and significant clusters feature. Panel **a**-**c**. Respiratory drive was measured by esophageal pressure drop during the first 100 msec of an end-expiratory occlusion (P0.1), respiratory effort by esophageal inspiratory pressure swings (∆Pes), and lung stress by dynamic transpulmonary pressure variations (∆Plung). Panel **d**-**e**. The personalised PEEP assigned by integrating EIT and transpulmonary pressure data across the whole trial and the intensive care unit (ICU) and length of stay were compared between the 3 clusters identified at PEEPlow. Panel **f**. Each EIT variable was tested for differences between clusters by bonferroni-corrected ANOVA/Kruskal-Wallis tests, significant features at PEEPlow are here represented with their *p*-values and intra-class correlation coefficients (ICC)

Heatmaps of EIT-based physiological variables at each PEEP level are shown in Fig. 1h. Stopping rules indicated most frequently two or three as the optimal number of clusters (Table S3). Silhouette plots were drawn for both options, showing fewer misclassification at PEEPlow by using three clusters (Fig. 2). To our knowledge, there is no clear-cut rule for naming clusters. Based on discussion between authors on heatmap visualization and on the main variables that characterised the three clusters (Fig. 3f; f, Table S4), these were named *unmatched V’/Q*, *mismatched V’/Q* and *inhomogeneous ventilation.* Indeed, the former two were mainly characterized by higher V’/Q mismatch, with variables more precisely defined in the first (e.g. dead space, shunt) and mainly indices of V’/Q inhomogeneity in the second The last, instead, seemed to be predominantly characterised by ventilation EIT data with little contribution of perfusion analysis.

At PEEPlow, respiratory drive, as measured by the occlusion esophageal pressure change during the first 100 msec (P0.1), was highest in the unmatched V’/Q cluster, lower in the mismatched V’/Q and minimum in the inhomogeneous ventilation clusters (*p* = 0.045, Fig. 3a). Respiratory effort, as assessed by the inspiratory esophageal pressure swings (∆Pes), was higher in the unmatched V’/Q and mismatched V’/Q clusters and lower in the inhomogeneous ventilation cluster (*p* = 0.021, Fig. 3b). Finally, dynamic lung stress, measured by transpulmonary pressure swings, was higher in the unmatched and mismatched clusters, but the difference did not reach statistical significance (*p* = 0.063, Fig. 3c). Interestingly, at PEEPintermediate and PEEPhigh (Fig. 3a-c), differences between the three sub-phenotypes seemed to disappear, while higher transpulmonary pressure values were observed in the inhomogeneous ventilation cluster at PEEPhigh (*p* = 0.047). (*p* = 0.047). Thus, EITomics could identify physiological cluster of ARDS with increased respiratory drive and effort at lower PEEP, potentially leading to higher risk of P-SILI (patient self-inflicted lung injury). Higher PEEP limited the respiratory drive of the unmatched V’/Q cluster, potentially suggesting benefits of higher personalized PEEP. Application of a novel EIT method [4, 19] balancing lung overdistension and collapse during spontaneous breathing, precisely selected higher personalised PEEP level for the unmatched V’/Q cluster, as compared to the other two (*p* = 0.025, Fig. 3d). More intense respiratory drive in the unmatched V’/Q cluster was also associated to longer ICU length of stay (*p* = 0.015, Fig. 3e), suggesting a causal link between increased respiratory drive and effort and worse clinical outcome.

Finally, to identify the main EIT variables differentiating clusters at PEEPlow, one-way ANOVA or corresponding non-parametric tests were applied to each of the 180 variables (Fig. 3f, Table S4). After Bonferroni correction, four EIT variables were found to be most relevant among all those used for clustering: dead space fraction (p < 0.001), ventilation to units characterised by high V’/Q ratio (*p* < 0.01), perfusion to units with normal V’/Q ratio (*p* < 0.01) and dorsal wasted perfusion (*p* < 0.01). These data further underline the physiological relevance of regional V’/Q mismatch as a determinant of increased respiratory drive and effort [22] and of worse clinical outcome [9].

## Discussion

EIT is a non-invasive, bedside, radiation-free lung imaging technique that is increasingly used for physiological characterization of ARDS and to guide personalised treatments [4, 5, 8]. Studies using EIT mainly focused on a small number of variables, either assessing regional respiratory mechanics [7] or ventilation and perfusion distribution [23, 24] or V’/Q mismatch [9]. However, physiology of ARDS is too complex and heterogenous to be comprehensively defined by a limited set of physiological data. Simultaneous approach to multiple biochemical, clinical and radiological variables by latent class analysis [25] or, more recently, by artificial intelligence [26] showed promising results for identification of physiologically and clinically relevant sub-phenotypes of ARDS, albeit with the key limitation of having limited potential for bedside translatability.

The present study used a comprehensive simultaneous approach to analyse 180 EIT variables, covering all domains of its applications, and identified 3 ARDS clusters. The analysis performed here could be easily implemented within commercial bedside EIT monitors and didn’t require any specific intervention, apart from recordings of EIT data during representative tidal breathing. We also selected hierarchical clustering as a simple to understand and readily available clustering methodology commonly used in biomedical sciences, that could be implemented in any laboratory with minimal statistical and computational background [13, 14]. EIT could represent the ideal tool to translate an in-depth, multi-dimensional, *physiomic* approach to the bedside care of ARDS.

The correlation between lung ventilation-perfusion mismatch and increased respiratory drive, and the one between increased drive and effort and worse clinical outcomes have recently been described in explorative clinical studies [22, 27]. Data from the present study further suggest that *a.* larger V’/Q defects (especially towards the dead space - high V’/Q mismatch types) could generate higher respiratory drive by multiple mechanisms (e.g., poor carbon dioxide clearance, lung collapse, etc.) and *b.* that higher drive could, in turn, increase lung stress causing additional P-SILI and prolonged ventilator dependency. While the link between lower PEEP and higher drive in hypoxemic intubated patients has been suggested [22] recent studies showed a more complex interaction, with some patients decreasing effort at lower PEEP [4]. A noveltyof our study was to identify, by EIT monitoring only, a cluster of patients with higher drive at lower PEEP and benefiting from increased PEEP; as well as two other patient clusters in whom drive was not affected by lower PEEP.

It is of course possible that the choice of the variables to include in the clustering process may have influenced our results. Any choice here could be debated and, ultimately, is dependent on the experimenter’s own background. We followed the principle that every EIT variable routinely measured in our laboratory for this type of patients should be included. This involved a certain degree of redundancy, especially after splitting each variable across four regions of interest (ROI), but redundancy and subtle differences in molecular functions lye at the basis of widely accepted biologic -omics analyses.

The main limitation of our study is its sample size, implying that this should be considered a proof of concept rather than a definitive assessment of spontaneously breathing ARDS patient clusters. Additionally, it is important to note that hierarchical clustering, by design, will always produce a clustering partition of the dataset. However, for this very reason, the technique is particularly well suited for an exploratory analysis like ours. More importantly, our agglomerative coefficients, along with external validation through the comparison of respiratory drive and effort between clusters, suggest that a genuine clustering structure is present in our data, warranting further evaluation.

Another limitation is the use of EIT pulsatile signal as a proxy for perfusion. It is well-known that paradoxical effects may appear in collapsed lung regions, where local perfusion is reduced, while pulsatility may be increased [28, 29]. However, we recently published an analysis of V’/Q matching based on pulsatility with very high accuracy [30] and attempts to obtain lung perfusion by indicator-based EIT data during uninterrupted spontaneous breathing are still in their prime [11] and may be prone to even less accuracy. Since our study was primarily hypothesis-generating rather than confirmatory, we chose to include the pulsatile analysis as a first step to determine its value in this context. While our results seem promising and generate interesting perspectives for assessing V’/Q mismatch in this type of patients, the effects of changing PEEP on pulsatility during spontaneous breathing are largely unknown and warrant caution in the interpretation of our results.

## Conclusions

Omics approach to physiological data at the bedside could represent a novel method to dissect the complexity and the heterogeneity of ARDS, which could facilitate predictive enrichment of randomised trials and discovery of specific personalised treatments.

## Electronic supplementary material

Below is the link to the electronic supplementary material.

Supplementary Material 1

## Data Availability

The datasets used and/or analysed during the current study are available from the corresponding author on reasonable request.

## Abbreviations

AC: Agglomerative coefficient
AGNES: Agglomerative nesting
ANOVA: Analysis of variance
ARDS: Acute respiratory distress syndrome
cmH2O: Centimetres of water
EIT: Electrical impedance tomography
FiO2: Fraction of inspired oxygen
HSD: Honestly significant difference
ICC: Intra–class correlation coefficient
PCA: Principal component analysis
PEEP: Positive end–expiratory pressure
PEEPeit: PEEP balancing overdistension and collapse obtained by EIT and esophageal pressure
PEEPhigh: PEEP 16–18 cmH2O
PEEPintermediate: PEEP 10–12–14 cmH2O
PEEPlow: PEEP 16–18 cmH2O
Pes: Esophageal pressure
Plung: Transpulmonary pressure
P-SILI: Patient self–inflicted lung injury
V’/Q: Ventilation/perfusion
