# Supplementary material for: Omics approach to chest electrical impedance tomography reveals physiological cluster of ARDS characterised by increased respiratory drive and effort
Source: Ann Intensive Care. 2025 Jul 8;15:90. doi: 10.1186/s13613-025-01514-3 (PMC12234929; doi:10.1186/s13613-025-01514-3)
Supplement: Supplementary file 1 — Supplementary Material 1 [file 13613_2025_1514_MOESM1_ESM.docx]

**Omics approach to chest Electrical Impedance Tomography reveals physiological cluster of ARDS characterised by increased respiratory drive and effort**

Tommaso Mauri^1,2^, Marco Leali^1^, Elena Spinelli^2^, Gaetano Scaramuzzo^3,4^, Massimo Antonelli^5,6^, Domenico L. Grieco^5,6^, Savino Spadaro^3,4^ Giacomo Grasselli^1,2^

1 Department of Pathophysiology and Transplantation, University of Milan, Milan, Italy; 2 Department of Emergency, Foundation IRCCS Ca' Granda Maggiore Policlinico Hospital, Milan, Italy; 3 Department of Translational Medicine, University of Ferrara, Ferrara, Italy; 4 Intensive Care Unit, Department of Morphology, Surgery and Experimental Medicine, Sant’Anna University Hospital, Ferrara, Italy; 5 Department of Emergency, Intensive Care Medicine and Anesthesia, Fondazione Policlinico Universitario A. Gemelli IRCCS, Rome, Italy; 6 Department of Anesthesiology and Intensive Care Medicine, Catholic University of the Sacred Heart, Rome, Italy.

**Supplement**

**Supplementary Methods**

Agglomerative coefficient (AC). The agglomerative coefficient (AC) was used to measure the strength of the obtained clustering structures. The AC is a dimensionless coefficient lying in the 0-1 interval and is part of the output of the agglomerative nesting (AGNES) algorithm. It approaches 1 as the between-cluster distances at the higher nodes in the dendrogram increases, as compared to the within-cluster distances at the lower nodes. It can be used to compare clustering results, as long as there are no relevant changes in the number of clustered units^13^. As two patients lacked data at the highest PEEP levels (16, 18 cmH2O), we kept these two levels separated from the others in the sensitivity analysis, to avoid missingness issues.

Stopping rules. More than thirty stopping rules have been proposed, which may aid in determining the best number of clusters after hierarchical clustering has been applied^15,16^. Five indices have been proposed due to their performance in simulations of ideal data^15^, while another five indices have been recommended for noisy data, as commonly found in the biomedical field and in our study^16^. Due to the highly correlated nature of some of our variables, methods based on the covariance matrix could not be applied, so that only three (Calinski & Harabasz, C-Index, Gamma statistic) and one (Ball & Hall) of the two groups of indices, respectively, could be calculated. The majority rule was applied for selecting the optimal number of clusters, assuming that the same clustering structure was to be found across all PEEP levels^17^. Obtained clusters were further inspected with the aid of silhouette plots^18^. The maximum number of clusters for testing was determined by the square root of the number of patients ($\sqrt{30} \approx5$) as a rule of thumb.

**Supplementary Table S1 | Complete list of EIT variables.** CO – cardiac output; CoP – center of perfusion; CoV – center of ventilation; CV – coefficient of variation; d/v – dorsal/ventral axis; EELI – end expiratory lung impedance; EXP – expiration; GI – global inhomogeneity index; GLI – GI times LI; I:E – inspiratory to expiratory time ratio; INSP – inspiration; LI – local inhomogeneity index; logSD – logarithmic standard deviation; MEF – minimal expiratory flow; MIF – maximal inspiratory flow MV – minute volume; PERF – perfusion; px – pixel; r/l – right/left axis; RR – respiratory rate; Te – expiratory time; Ti - inspiratory time; TIV – tidal impedance variation; VENT – ventilation; V’/Q – ventilation/perfusion ratio; x-coord – x-coordinate; y-coord – y-coordinate

| CoP (x-coord) | Low V'/Q [px] (%) | Ti |
| --- | --- | --- |
| CoP (y-coord) | Low V'/Q [px] (%dorsal) | Time Difference EXP |
| CoV (d/v) | Low V'/Q [px] (%left) | Time Difference EXP (dorsal) |
| CoV (r/l) | Low V'/Q [px] (%right) | Time Difference EXP (left) |
| Dead Space [px] (%) | Low V'/Q [px] (%ventral) | Time Difference EXP (right) |
| Dead Space [px] (%dorsal) | Low V'/Q [ventilation] (%) | Time Difference EXP (ventral) |
| Dead Space [px] (%left) | Low V'/Q [ventilation] (%dorsal) | Time Difference INSP |
| Dead Space [px] (%right) | Low V'/Q [ventilation] (%left) | Time Difference INSP (dorsal) |
| Dead Space [px] (%ventral) | Low V'/Q [ventilation] (%right) | Time Difference INSP (left) |
| Dead Space [ventilation] (%) | Low V'/Q [ventilation] (%ventral) | Time Difference INSP (right) |
| Dead Space [ventilation] (%dorsal) | MEF (dorsal) | Time Difference INSP (ventral) |
| Dead Space [ventilation] (%left) | MEF (left) | TIV (%dorsal) |
| Dead Space [ventilation] (%right) | MEF (right) | TIV (%left) |
| Dead Space [ventilation] (%ventral) | MEF (ventral) | TIV (%right) |
| Dead Space Fraction (%) | MIF (dorsal) | TIV (%ventral) |
| Dead Space Fraction (%dorsal) | MIF (left) | V'/Q PERF |
| Dead Space Fraction (%left) | MIF (right) | V'/Q PERF (dorsal) |
| Dead Space Fraction (%right) | MIF (ventral) | V'/Q PERF (left) |
| Dead Space Fraction (%ventral) | Normal V'/Q [perfusion] (%) | V'/Q PERF (right) |
| EELI (%dorsal) | Normal V'/Q [perfusion] (%dorsal) | V'/Q PERF (ventral) |
| EELI (%left) | Normal V'/Q [perfusion] (%left) | V'/Q VENT |
| EELI (%right) | Normal V'/Q [perfusion] (%right) | V'/Q VENT (dorsal) |
| EELI (%ventral) | Normal V'/Q [perfusion] (%ventral) | V'/Q VENT (left) |
| Estimated MV/CO (ml/ml) | Normal V'/Q [px] (%) | V'/Q VENT (right) |
| Global Pendelluft [Sang 2020]^1^ | Normal V'/Q [px] (%dorsal) | V'/Q VENT (ventral) |
| Global Pendelluft [Sang 2020] (dorsal) | Normal V'/Q [px] (%left) | Ventilated Only px (%) |
| Global Pendelluft [Sang 2020] (left) | Normal V'/Q [px] (%right) | Ventilated Only px (%dorsal) |
| Global Pendelluft [Sang 2020] (right) | Normal V'/Q [px] (%ventral) | Ventilated Only px (%left) |
| Global Pendelluft [Sang 2020] (ventral) | Normal V'/Q [ventilation] (%) | Ventilated Only px (%right) |
| High V'/Q [perfusion] (%) | Normal V'/Q [ventilation] (%dorsal) | Ventilated Only px (%ventral) |
| High V'/Q [perfusion] (%dorsal) | Normal V'/Q [ventilation] (%left) | Ventilation CV |
| High V'/Q [perfusion] (%left) | Normal V'/Q [ventilation] (%right) | Ventilation CV (dorsal) |
| High V'/Q [perfusion] (%right) | Normal V'/Q [ventilation] (%ventral) | Ventilation CV (left) |
| High V'/Q [perfusion] (%ventral) | Occult Pendelluft [Cornejo 2022]^2^ | Ventilation CV (right) |
| High V'/Q [px] (%) | Perfused Only (%ventral) | Ventilation CV (ventral) |
| High V'/Q [px] (%dorsal) | Perfused Only px (%) | Ventilation GI |
| High V'/Q [px] (%left) | Perfused Only px (%dorsal) | Ventilation GI (dorsal) |
| High V'/Q [px] (%right) | Perfused Only px (%left) | Ventilation GI (left) |
| High V'/Q [px] (%ventral) | Perfused Only px (%right) | Ventilation GI (right) |
| High V'/Q [ventilation] (%) | Perfusion (%dorsal) | Ventilation GI (ventral) |
| High V'/Q [ventilation] (%dorsal) | Perfusion (%left) | Ventilation GLI |
| High V'/Q [ventilation] (%left) | Perfusion (%right) | Ventilation GLI (dorsal) |
| High V'/Q [ventilation] (%right) | Perfusion (%ventral) | Ventilation GLI (left) |
| High V'/Q [ventilation] (%ventral) | RR | Ventilation GLI (right) |
| I:E | Shunt [perfusion] (%) | Ventilation GLI (ventral) |
| logSD PERF | Shunt [perfusion] (%dorsal) | Ventilation LI |
| logSD PERF (dorsal) | Shunt [perfusion] (%left) | Ventilation LI (dorsal) |
| logSD PERF (left) | Shunt [perfusion] (%right) | Ventilation LI (left) |
| logSD PERF (right) | Shunt [perfusion] (%ventral) | Ventilation LI (right) |
| logSD PERF (ventral) | Shunt [px] (%) | Ventilation LI (ventral) |
| logSD VENT | Shunt [px] (%dorsal) | Wasted Perfusion (%) |
| logSD VENT (dorsal) | Shunt [px] (%left) | Wasted Perfusion (%dorsal) |
| logSD VENT (left) | Shunt [px] (%right) | Wasted Perfusion (%left) |
| logSD VENT (right) | Shunt [px] (%ventral) | Wasted Perfusion (%right) |
| logSD VENT (ventral) | Shunt Fraction (%) | Wasted Perfusion (%ventral) |
| Low V'/Q [perfusion] (%) | Shunt Fraction (%dorsal) | Wasted Ventilation (%) |
| Low V'/Q [perfusion] (%dorsal) | Shunt Fraction (%left) | Wasted Ventilation (%dorsal) |
| Low V'/Q [perfusion] (%left) | Shunt Fraction (%right) | Wasted Ventilation (%left) |
| Low V'/Q [perfusion] (%right) | Shunt Fraction (%ventral) | Wasted Ventilation (%right) |
| Low V'/Q [perfusion] (%ventral) | Te | Wasted Ventilation (%ventral) |
| 1. Sang, L. *et al.* Qualitative and quantitative assessment of pendelluft: a simple method based on electrical impedance tomography. *Annals of Translational Medicine* **8**, 1216–1216 (2020). 2. Cornejo, R. A. *et al.* Inflammatory biomarkers and pendelluft magnitude in ards patients transitioning from controlled to partial support ventilation. *Sci Rep* **12**, 20233 (2022). | | |

**Supplementary Table S2 | Sensitivity analysis.** The agglomerative coefficient (AC) is shown, which measures cluster strength and varies across different linkage methods (columns) and different PEEP step combinations (rows). The highest mean AC was found for Ward’s linkage criterion and for PEEP level averaging into three steps.

| **AC** | **average** | **single** | **complete** | **Ward’s** | **weighted** |
| --- | --- | --- | --- | --- | --- |
| *Single PEEP levels* | |  |  |  |  |
| 4 | 0.45 | 0.34 | 0.54 | 0.67 | 0.47 |
| 6 | 0.494 | 0.375 | 0.56 | 0.666 | 0.543 |
| 8 | 0.387 | 0.312 | 0.517 | 0.694 | 0.42 |
| 10 | 0.425 | 0.291 | 0.541 | 0.642 | 0.455 |
| 12 | 0.379 | 0.191 | 0.528 | 0.739 | 0.406 |
| 14 | 0.381 | 0.228 | 0.55 | 0.699 | 0.427 |
| 16 | 0.448 | 0.281 | 0.562 | 0.68 | 0.444 |
| 18 | 0.433 | 0.275 | 0.581 | 0.681 | 0.471 |
| **average** | 0.425 | 0.287 | 0.548 | 0.684 | 0.454 |
| *Two PEEP levels* | |  |  |  |  |
| 4+6 | 0.493 | 0.365 | 0.583 | 0.655 | 0.565 |
| 8+10 | 0.403 | 0.333 | 0.523 | 0.667 | 0.436 |
| 12+14 | 0.393 | 0.208 | 0.555 | 0.734 | 0.421 |
| 16+18 | 0.459 | 0.281 | 0.576 | 0.69 | 0.499 |
| **average** | 0.437 | 0.297 | 0.559 | 0.687 | 0.48 |
| *Three PEEP levels* | |  |  |  |  |
| 4+6+8 | 0.476 | 0.404 | 0.575 | **0.647** | 0.504 |
| 10+12+14 | 0.379 | 0.202 | 0.54 | **0.725** | 0.418 |
| 16+18 | 0.459 | 0.281 | 0.576 | **0.69** | 0.499 |
| **average** | 0.438 | 0.296 | 0.564 | **0.688** | 0.473 |

**Supplementary Table S3 | Optimal number of clusters according to four widely used stopping rules ^12,13^** (see Methods for further details). These were calculated for the three PEEP steps used in our study and for all PEEP steps averaged into one. The majority rule was used to determine the optimal number of clusters, which was kept the same for all PEEP steps.

| **Step** | **Calinski & Harabasz** | **C-Index** | **Gamma statistic** | **Ball & Hall** |
| --- | --- | --- | --- | --- |
| PEEPlow | 2 | 2 | 5 | 3 |
| PEEPintermediate | 2 | 3 | 5 | 3 |
| PEEPhigh | 2 | 4 | 5 | 3 |
| all steps averaged | 2 | 3 | 5 | 3 |

**Supplementary Table S4** | **Significant features differentiating the three clusters at each PEEP level.** All features resulting in significant p-value after Bonferroni correction with an alpha 0.99 confidence level in at least one PEEP step have been shown across all steps.

|  | *PEEPlow* | | | | | | |
| --- | --- | --- | --- | --- | --- | --- | --- |
|  | **ALL**  **(n=30)** | **UNMATCHED**  **(n=15)** | **MISMATCHED**  **(n=9)** | **INHOMOG. VENTILATION**  **(n=6)** | **p** | **Bonferroni correction** | **ICC** |
| CoV (d/v) | 17.74 ± 1.47 | 18.37 ± 1.4 | 17.11 ± 1.42 | 17.11 ± 1.19 | 0.058 | 1 | 0.23 |
| Ventilation LI (ventral) | 0.09 ± 0.02 | 0.10 ± 0.02 | 0.08 ± 0.02 | 0.08 ± 0.01 | 0.059 | 1 | 0.22 |
| Ventilation GLI (ventral) | 0.03 ± 0.01 | 0.03 ± 0.01 | 0.03 ± 0.01 | 0.03 ± 0 | 0.022 | 1 | 0.31 |
| Dead Space Fraction (%) | 72.2 ± 15.96 | 85.2 ± 7.38 | 60.73 ± 12.11 | 56.9 ± 8.08 | <0.001 | <0.001 | 0.8 |
| High V'/Q [px] (%) | 0.4 ± 0.16 | 0.49 ± 0.14 | 0.38 ± 0.13 | 0.22 ± 0.05 | <0.001 | 0.306 | 0.55 |
| High V'/Q [px] (%right) | 0.45 ± 0.21 | 0.54 ± 0.19 | 0.46 ± 0.16 | 0.2 ± 0.07 | <0.001 | 0.455 | 0.53 |
| High V'/Q [px] (%dorsal) | 0.29 ± 0.19 | 0.35 ± 0.19 | 0.31 ± 0.19 | 0.12 ± 0.06 | 0.029 | 1 | 0.29 |
| High V'/Q [ventilation] (%) | 59.06 ± 20.99 | 72.46 ± 15.7 | 55.41 ± 13.8 | 31.06 ± 7.48 | <0.001 | 0.003 | 0.71 |
| High V'/Q [ventilation] (%dorsal) | 45.7 ± 25.43 | 57.96 ± 23.5 | 43.93 ± 21.52 | 17.73 ± 8.24 | 0.002 | 1 | 0.48 |
| High V'/Q [ventilation] (%ventral) | 64.12 ± 24.06 | 77.68 ± 15.9 | 56.83 ± 27.19 | 41.17 ± 13.73 | 0.002 | 1 | 0.49 |
| High V'/Q [perfusion] (%dorsal) | 34.2 ± 22.02 | 42.09 ± 22.94 | 33.62 ± 19.63 | 15.35 ± 10.41 | 0.036 | 1 | 0.27 |
| Normal V'/Q [perfusion] (%) | 24.38 ± 11.03 | 15.84 ± 6.73 | 34.25 ± 7.3 | 30.94 ± 6.89 | <0.001 | <0.001 | 0.75 |
| Wasted Perfusion (%) | 49.71 ± 20.38 | 63 ± 17.4 | 40.76 ± 14.03 | 29.9 ± 10.01 | <0.001 | 0.089 | 0.6 |
| Wasted Perfusion (%left) | 51.68 ± 23.03 | 64.06 ± 18.49 | 46.71 ± 19.96 | 28.21 ± 17.81 | 0.002 | 1 | 0.49 |
| Wasted Perfusion (%dorsal) | 52.91 ± 22.53 | 69.69 ± 15.46 | 37.54 ± 12.03 | 34.03 ± 18.57 | <0.001 | 0.005 | 0.7 |
| Perfused Only px (%dorsal) | 10.59 ± 7.72 | 12.93 ± 8.02 | 10.53 ± 7.32 | 4.8 ± 4.78 | 0.089 | 1 | 0.18 |
| logSD PERF | 0.44 (0.29 - 0.49) | 0.49 (0.42 - 0.54) | 0.35 (0.29 - 0.45) | 0.27 (0.25 - 0.37) | 0.003 | 1 | 0.45 |
| logSD PERF (dorsal) | 0.4 ± 0.15 | 0.46 ± 0.16 | 0.36 ± 0.08 | 0.29 ± 0.13 | 0.026 | 1 | 0.3 |
|  | ***PEEPintermediate*** | | | | | | |
|  | **ALL**  **(n=30)** | **UNMATCHED**  **(n=7)** | **MISMATCHED**  **(n=11)** | **INHOMOG. VENTILATION**  **(n=12)** | **p** | **Bonferroni correction** | **ICC** |
| CoV (d/v) | 16.82 ± 1.51 | 18.79 ± 0.59 | 15.57 ± 1.26 | 16.81 ± 0.58 | <0.001 | <0.001 | 0.75 |
| Ventilation LI (ventral) | 0.08 ± 0.02 | 0.11 ± 0.01 | 0.06 ± 0.01 | 0.08 ± 0.01 | <0.001 | <0.001 | 0.81 |
| Ventilation GLI (ventral) | 0.03 (0.02 - 0.03) | 0.03 (0.03 - 0.04) | 0.02 (0.02 - 0.02) | 0.03 (0.02 - 0.03) | <0.001 | 0.002 | 0.69 |
| Dead Space Fraction (%) | 72.11 ± 20.25 | 89.36 ± 7.55 | 83.56 ± 7.59 | 51.56 ± 14.71 | <0.001 | <0.001 | 0.79 |
| High V'/Q [px] (%) | 0.43 ± 0.16 | 0.51 ± 0.08 | 0.56 ± 0.08 | 0.26 ± 0.1 | <0.001 | <0.001 | 0.79 |
| High V'/Q [px] (%right) | 0.47 ± 0.21 | 0.56 ± 0.08 | 0.64 ± 0.13 | 0.27 ± 0.15 | <0.001 | <0.001 | 0.74 |
| High V'/Q [px] (%dorsal) | 0.38 ± 0.23 | 0.29 ± 0.09 | 0.62 ± 0.13 | 0.21 ± 0.15 | <0.001 | <0.001 | 0.76 |
| High V'/Q [ventilation] (%) | 60.92 ± 23.57 | 80.83 ± 12.67 | 75.17 ± 10.44 | 36.24 ± 12.59 | <0.001 | <0.001 | 0.83 |
| High V'/Q [ventilation] (%dorsal) | 54.8 ± 28.04 | 64.36 ± 23.39 | 76.61 ± 12.65 | 29.24 ± 19.55 | <0.001 | 0.002 | 0.68 |
| High V'/Q [ventilation] (%ventral) | 73.3 (41.1 - 80.9) | 82.5 (79.6 - 95.9) | 77.0 (60.2 - 82.4) | 40.7 (31.9 - 45.1) | <0.001 | 0.003 | 0.67 |
| High V'/Q [perfusion] (%dorsal) | 38.7 (17.3 - 67.8) | 27.3 (24.9 - 39.7) | 69.2 (67.3 - 83.3) | 15.1 (6.6 - 30.0) | <0.001 | 0.001 | 0.7 |
| Normal V'/Q [perfusion] (%) | 23.52 ± 13.49 | 11.44 ± 7.19 | 21.28 ± 9.26 | 32.62 ± 13.63 | 0.001 | 0.54 | 0.46 |
| Wasted Perfusion (%) | 49.04 ± 24.39 | 74.83 ± 18.33 | 54.94 ± 19.62 | 28.59 ± 10.95 | <0.001 | 0.004 | 0.67 |
| Wasted Perfusion (%left) | 49.89 ± 25.55 | 77.45 ± 19.11 | 55.92 ± 15.78 | 28.27 ± 16.32 | <0.001 | 0.002 | 0.68 |
| Wasted Perfusion (%dorsal) | 52.54 ± 25.56 | 81.56 ± 15.68 | 56.11 ± 22.49 | 32.34 ± 11.61 | <0.001 | 0.005 | 0.66 |
| Perfused Only px (%dorsal) | 8.37 ± 8.06 | 20.27 ± 6.7 | 4.56 ± 3.95 | 4.93 ± 3.86 | <0.001 | <0.001 | 0.76 |
| logSD PERF | 0.39 ± 0.14 | 0.55 ± 0.05 | 0.39 ± 0.12 | 0.3 ± 0.09 | <0.001 | 0.03 | 0.6 |
| logSD PERF (dorsal) | 0.36 ± 0.16 | 0.57 ± 0.06 | 0.29 ± 0.13 | 0.3 ± 0.1 | <0.001 | 0.005 | 0.66 |
|  | ***PEEPhigh*** | | | | | | |
|  | **ALL**  **(n=28)** | **UNMATCHED**  **(n=6)** | **MISMATCHED**  **(n=15)** | **INHOMOG. VENTILATION**  **(n=7)** | **p** | **Bonferroni correction** | **ICC** |
| CoV (d/v) | 16.05 ± 1.45 | 17.92 ± 0.72 | 15.2 ± 1.19 | 16.27 ± 0.63 | <0.001 | 0.017 | 0.7 |
| Ventilation LI (ventral) | 0.07 ± 0.02 | 0.1 ± 0.01 | 0.06 ± 0.02 | 0.07 ± 0.01 | <0.001 | 0.091 | 0.64 |
| Ventilation GLI (ventral) | 0.02 ± 0.01 | 0.03 ± 0 | 0.02 ± 0.01 | 0.02 ± 0 | <0.001 | 0.15 | 0.62 |
| Dead Space Fraction (%) | 67.2 ± 24.28 | 89.6 ± 7.28 | 73.97 ± 10.81 | 33.48 ± 19.81 | <0.001 | <0.001 | 0.84 |
| High V'/Q [px] (%) | 0.4 ± 0.17 | 0.49 ± 0.1 | 0.46 ± 0.1 | 0.17 ± 0.11 | <0.001 | 0.001 | 0.77 |
| High V'/Q [px] (%right) | 0.43 ± 0.2 | 0.54 ± 0.1 | 0.51 ± 0.14 | 0.15 ± 0.11 | <0.001 | 0.001 | 0.77 |
| High V'/Q [px] (%dorsal) | 0.39 ± 0.23 | 0.37 ± 0.13 | 0.54 ± 0.17 | 0.1 ± 0.07 | <0.001 | 0.002 | 0.76 |
| High V'/Q [ventilation] (%) | 57.44 ± 25.23 | 80.37 ± 12.94 | 64.84 ± 12.97 | 21.95 ± 14.18 | <0.001 | <0.001 | 0.84 |
| High V'/Q [ventilation] (%dorsal) | 54.57 ± 28.6 | 72.47 ± 19.5 | 66.61 ± 16.29 | 13.42 ± 9.29 | <0.001 | <0.001 | 0.83 |
| High V'/Q [ventilation] (%ventral) | 56.67 ± 25.72 | 84.35 ± 10.27 | 58.04 ± 19.87 | 30.01 ± 19.15 | <0.001 | 0.035 | 0.68 |
| High V'/Q [perfusion] (%dorsal) | 44.07 ± 28.28 | 44.93 ± 18.42 | 60.33 ± 22.01 | 8.47 ± 6.66 | <0.001 | 0.007 | 0.73 |
| Normal V'/Q [perfusion] (%) | 26.53 ± 16.35 | 10.12 ± 5.87 | 25.94 ± 10.37 | 41.87 ± 19.47 | <0.001 | 0.289 | 0.59 |
| Wasted Perfusion (%) | 48.71 ± 26.21 | 78.25 ± 14.58 | 49.86 ± 21.16 | 20.94 ± 9.7 | <0.001 | 0.012 | 0.71 |
| Wasted Perfusion (%left) | 49.5 ± 27.79 | 80.87 ± 17.98 | 50.9 ± 21.42 | 19.59 ± 10.21 | <0.001 | 0.009 | 0.72 |
| Wasted Perfusion (%dorsal) | 48.41 ± 30.49 | 86.09 ± 12.23 | 45.06 ± 26.81 | 23.27 ± 14.01 | <0.001 | 0.053 | 0.66 |
| Perfused Only px (%dorsal) | 7.25 ± 7.28 | 18.97 ± 5.31 | 4.36 ± 3.6 | 3.41 ± 3.11 | <0.001 | <0.001 | 0.84 |
| logSD PERF | 0.4 ± 0.15 | 0.56 ± 0.04 | 0.4 ± 0.11 | 0.23 ± 0.09 | <0.001 | 0.003 | 0.75 |
| logSD PERF (dorsal) | 0.35 ± 0.18 | 0.6 ± 0.06 | 0.3 ± 0.13 | 0.23 ± 0.11 | <0.001 | 0.002 | 0.76 |
